# Auto-CSC: A Transfer Learning Based Automatic Cell Segmentation and Count Framework

**DOI:** 10.34133/2022/9842349

**Published:** 2022-04-09

**Authors:** Guangdong Zhan, Wentong Wang, Hongyan Sun, Yaxin Hou, Lin Feng

**Affiliations:** ^1^Department of Mechanical Engineering & Automation, Beihang University, Beijing, China; ^2^Department of Diagnostic Ultrasound, Beijing Tongren Hospital, Capital Medical University, Beijing 100730, China; ^3^Beijing Advanced Innovation Center for Biomedical Engineering, Beihang University, Beijing 100083, China

## Abstract

Cell segmentation and counting play a very important role in the medical field. The diagnosis of many diseases relies heavily on the kind and number of cells in the blood. convolution neural network achieves encouraging results on image segmentation. However, this data-driven method requires a large number of annotations and can be a time-consuming and expensive process, prone to human error. In this paper, we present a novel frame to segment and count cells without too many manually annotated cell images. Before training, we generated the cell image labels on single-kind cell images using traditional algorithms. These images were then used to form the train set with the label. Different train sets composed of different kinds of cell images are presented to the segmentation model to update its parameters. Finally, the pretrained U-Net model is transferred to segment the mixed cell images using a small dataset of manually labeled mixed cell images. To better evaluate the effectiveness of the proposed method, we design and train a new automatic cell segmentation and count framework. The test results and analyses show that the segmentation and count performance of the framework trained by the proposed method equal the model trained by large amounts of annotated mixed cell images.

## 1. Introduction

The number and kinds of cells in the blood play an important role in disease diagnosis in clinical medicine [1]. In this process, the cell analyzer is used to count the number of cells according to their different physical properties [2]. However, this process can be complex and expensive.

The medical image field has been witnessing progressive advancements in recent decades. Cell segmentation is a popular research field in this context. Segmentation is to pixel-wise label the region of interest in an image. Cell images could be captured by connecting a high-definition camera to the microscope. For single-kind cell image segmentation, traditional methods are mainly based on threshold binarization or edge detection [3], and the watershed algorithm is typically used for overlapping cell images [4]. However, due to the different camera parameters and the complexity of the cell living environment, the above methods are inadequate for mixed cell images segmentation [5]. In recent years, convolution neural network (CNN) has been widely used in image classification [6], target detection [7], image denoising [8], semantic segmentation [9], and other tasks in the field of computer vision. For example, fully convolution networks are used to extract the characteristics of the cell images and properly classify them [10].

U-Net [11] is a fully convolutional network (FCN) semantic segmentation model with a contraction path to extract features and has an expansion path to localize the region of interest. The encoder and decoder, along with skip connections of U-Net, are proved to be more suitable for biomedical image processing. In [12], Zhou et al. presented UNet++, a nested U-Net architecture used for medical image segmentation. Their network semantically extracts similar feature maps for the encoder and decoder to improve the performance of the model. Arbelle et al. [13] proposed a network integrating convolutional long short-term memory (C-LSTM) with the U-Net. LSTM is used to analyze dynamic behavior, and U-Net is utilized to capture spatial properties of the data. Punn et al. [14] trained an inception U-Net architecture inspired by Google's inception architecture and the U-Net architecture for semantic segmentation. They replaced the convolution layers in the U-Net with inception layers to identify the nuclei in microscopy cell images.

Deep learning is a method that requires a lot of data to train models. To train such data-driven models, a huge amount of data with pixel-wise labels is required. However, manual labeling data, even with the assistance of experts, is a laborious and expensive process [15]. In this paper, we propose a new framework to train the U-Net model without too many manual annotations. The main contributions of this paper are as follows:

1. We propose an automatic and efficient method to segment single-kind cell images
2. Experiments demonstrate that the fine-tune U-Net model trained by the autolabeled single-kind-cell images offers similar performance with the model trained by the manual labels
3. The proposed framework is simple and effective to segment mixed-kind-cell images

## 2. Materials and Methods

**Figure 1:**
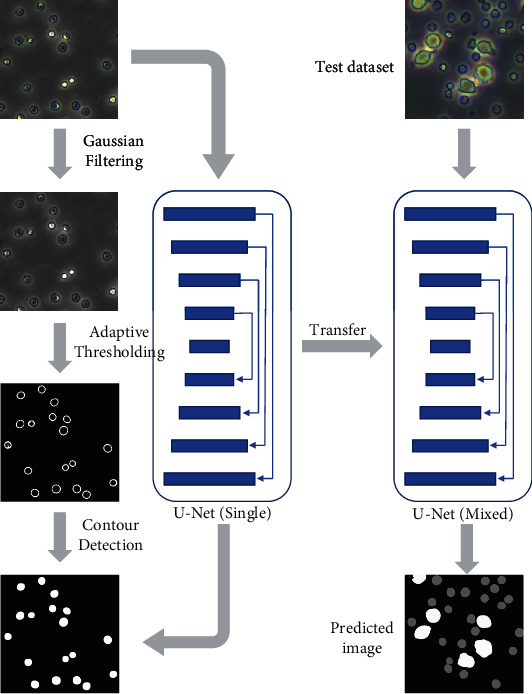
Flow diagram of auto-CSC.

In this paper, the training sets composed of single-kind cell images processed by preprocessing are presented to the U-Net model, and we fine-tuned the learned model by using manually annotated mixed-kind cell images to segment and count the mixed-kind cell images. This method is illustrated in Figure 1.

### 2.1. Preprocessing

We generated the cell image labels on single-kind cell images using traditional algorithms, including Gaussian filtering, adaptive thresholding, contour detection, and morphological processing.

#### 2.1.1. Gaussian Filtering

**Figure 2:**
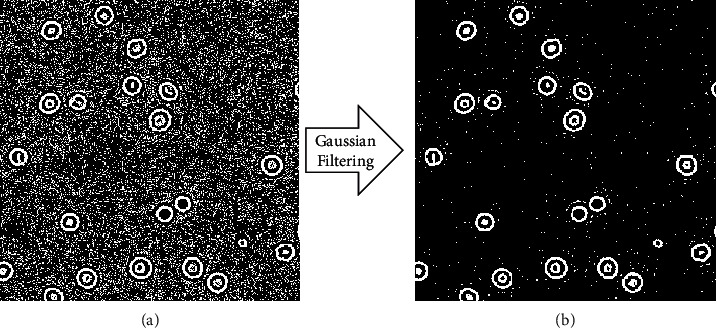
The result of (a) binarization original red blood cells (RBCs) image and (b) RBCs image processed by Gaussian filter.

Due to electromagnetic interference, a lot of noise is produced in the process of capturing cell images by electronic equipment. Filtering is a neighborhood operator, capable of removing noise and enhancing the image features we need. In this paper, we use a Gaussian filter to process the cell images and remove potential noises [16]. The reduction of noise could improve the accuracy of cell edge detection. We set the size of the Gaussian kernel to 3×3 and the Gaussian kernel standard deviation in the row and column direction to 0.8. .As shown Figure 2, the method is effective.

#### 2.1.2. Adaptive Thresholding

Binarization is an image segmentation algorithm based on thresholds. Firstly, the algorithm calculates one or more gray thresholds according to the gray level histograms of the image. Then, it compares the gray value of each pixel in the image with the threshold and finally divides the pixels into appropriate categories according to the comparison results [17]. Binarization is mainly divided into global thresholding [18] and adaptive thresholding. Global thresholding segments images according to a fixed threshold, while adaptive thresholding segments images according to the local feature of the image [19].

The gray value of different positions of the cell image will be discrepant due to the influence of the environment and the variant shooting conditions. We first scan the whole image with a sliding window (3×3 Gaussian kernel) and then calculate the threshold of each pixel in this window using the Gaussian function, according to the position of the pixel position. Finally, we segment the cell images with the threshold [3].

#### 2.1.3. Contour Detection

The Suzuki algorithm generates the closed outermost border without a parent border in the binary image, which is suitable for detecting the contour of the cells in the image [20]. Considering the influence of impurities or broken cells in the cell culture solution, we remove the boundaries of small areas in the gray image. However, this was only possible after finding the boundaries of all cells in the single-kind cell image.

**Figure 3:**
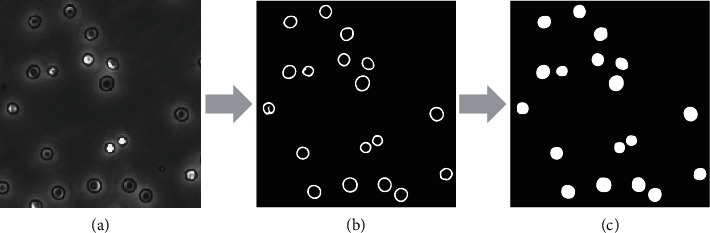
(a) original cell image, (b) the cell image processed by Suzuki algorithm, and (c) filled cell image (mask image).

Finally, we fill the detected contours to generate the mask images shown in Figure 3. The original single-kind cell images and the corresponding mask image (ground truth) form the training set.

#### 2.1.4. Morphological Processing

**Figure 4:**
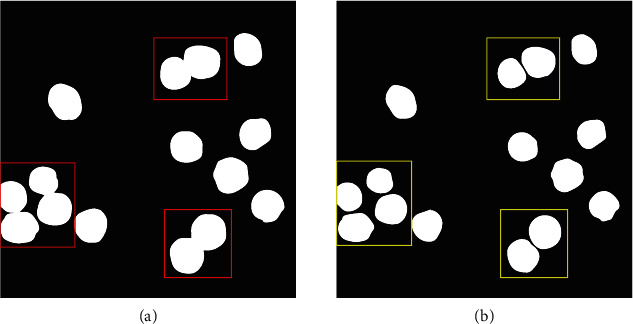
(a) Red boxes indicate adhesive macrophage, and (b) yellow boxes denote separated cell processed by the morphological processing.

Macrophages are a type of white blood cell of the immune system, and their main function is to engulf and digest cancer cells, microbes, cellular debris, and foreign substances. Some macrophages for their biological characteristic stick together as shown in Figure 4(a). As a result, macrophage images will be processed by dilation and erosion methods after contour detection. In this study, the convolution kernel size is set to 3×3, and the anchor is placed at the kernel center. This convolution kernel will slide along the macrophage image from left to right and from top to bottom. Then, it will calculate the product of the pixels at the corresponding position of the window and the convolution kernel. We select the minimum value as the pixel of the anchor position when eroding the image and the maximum value when dilating the image. That operation is formulated as follows:

(1) $$\begin{array}{c} \text{dst}\left(x,y\right)=\underset{\left(x^{\prime },y^{\prime }\right)\neq 0}{\min}\text{ src}\left(x+x^{\prime },y+y^{\prime }\right), \end{array}$$

(2) $$\begin{array}{c} \text{dst}\left(x,y\right)=\underset{\left(x^{\prime },y^{\prime }\right)\neq 0}{\max}\text{ src}\left(x+x^{\prime },y+y^{\prime }\right), \end{array}$$

where dst(*x*, *y*) denotes the image after a morphological operation, src denotes the original image, and (*x*′, *y*′) is the size of the convolution kernel.

Dilation will be taken after an erosion operation that would cause the macrophage area to narrow. As can be seen from Figure 4(b), the adhesive cells can be effectively separated after morphological processing.

### 2.2. U-Net

**Figure 5:**
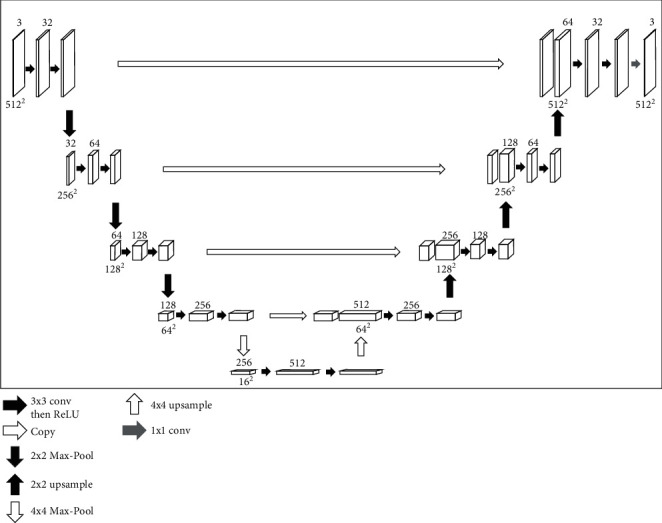
The architecture of U-Net (the input image is 512×512 pixels and 3 channels). The boxes denote the feature map. The arrows mean the different operations.

U-Net is an FCN for biomedical image segmentation trained with labeled images, and the name derives from the U-shaped structure. U-Net has been widely used in medical image segmentation since it was proposed in 2015 [11], and its effectiveness has been proved [12, 21]. The U-shaped design of the model makes full use of characteristic information at all levels of the image and has a significant effect on segmenting medical images with simple semantics and a single structure. U-Net consists of an encoding path on the left and a decoding path on the right, as shown in Figure 5.

There are three main modules of this U-shaped network: an encoder (downsampling) to capture the high-level abstract information for classifying the semantical meanings, a decoder (upsampling) to restore the resolution of the feature map, and a skip connection that can provide more characteristics for the decoder to reconstruct the fine details of the object. These unique structures make this network suitable for the simple semantic, fixed structure, and few number medical image dataset [22, 23].

Specifically, the network contains 19 convolution layers, 4 max-pooling layers, and 4 upsampling layers (nearest-neighbor interpolation). In this paper, the size of all convolution kernels is standard: 3×3. The strides and padding are 1 to avoid altering the image size after convolution. After two convolutions, a ReLU function is employed to generate nonlinear mapping, and a 2×2 max-pooling operation with stride 2 is conducted for downsampling. The decoding path replaces max-pooling with the nearest neighbor interpolation method to upsample the images. In the final layer, a 1×1 convolution is used to map the 32-channel feature map to 3 channels (the final predicted image). There is a skip connection between the same floor of the encoding and the decoding path. The bottom layer is 4×4 max-pooling and 4× scales upsample.

VGG16 is often used as the backbone of the object detection network for feature extraction. VGG16 consists of 13 convolutional layers, each followed by ReLU activation function, and 5 max-pooling operations, each reducing feature map by 2. All convolutional layers have 3×3 kernels. To construct U-Net, we remove the fully connected layers and max-pooling of VGG16 and use the first 12 pretrained convolution layers as the convolution layer of the encoder of the proposed U-Net to improve the ability of feature extraction. In order to construct the decoder of the proposed U-Net, we use convolution layer and nearest-neighbor interpolation to double the size of feature mapping and reduce the number of channels by half. Then, the output of upsampling is cascaded with the output of the corresponding part of the decoder. The feature map generated by convolution operation is processed to keep the number of channels the same as that in the symmetric encoder.

### 2.3. Dataset Description

**Figure 6:**
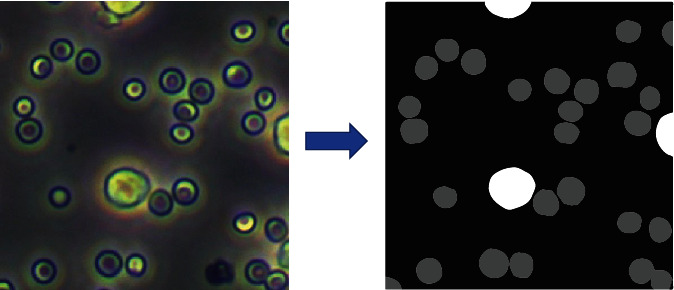
Manually annotated mixed cell images by several experts.

**Figure 7:**
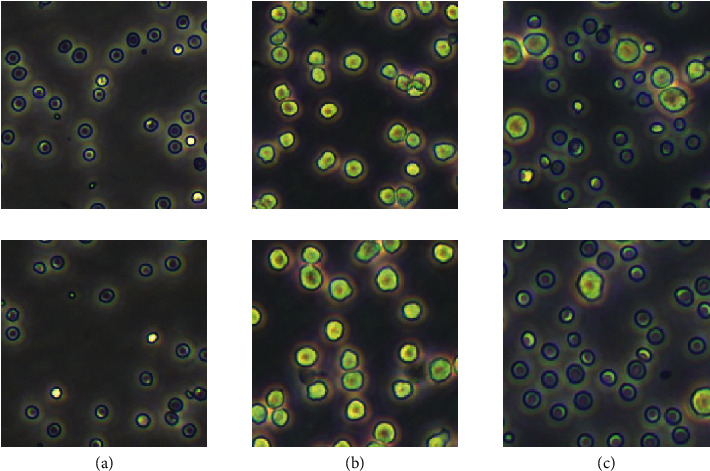
Illustration of the dataset used in this study: (a) RBCs, (b) macrophages, and (c) mixed-kind cells.

The red blood cells (RBCs) used in this research were collected from whole blood extracted from the tail tip of mice (KM mice, 8 weeks). The mouse mononuclear macrophage leukemia cell line (RAW264.7) was purchased from the Cell Resource Center, part of the Institute of Basic Medical Sciences of the Chinese Academy of Medical Sciences. Important and distinguishing features of RBCs are their evident discoid shape and smaller size (5-7 *μ*m approximately). RAW264.7 cells are adherent cells with an anomalous round morphology and a size distribution of 13-20 um. Both RBCs and RAW264.7 were cultured in high glucose Dulbecco's modified Eagle's medium (DMEM) (Hyclone). They were also supplemented with 10% (v/v) fetal bovine serum (FBS) (M6546-100 ml, Macklin) and kept at 37°C in a humidified atmosphere of 5% CO_2_. After counting the cells with a fully autocell analyzer (Bodboge), we proportionally mixed two different kinds of it: RBCs and macrophages. The dataset used in this paper consists of 1000 RBCs images, 1000 macrophage images, and 600 annotated mixed cell images. All images were captured by connecting a high-resolution camera (Camera: USB3.0 MicroUH1200, Ruizhi Image, China, Software: Digital-Camera 6.0) to an Olympus CKX53 microscope and cropped into 512×512 resolution from 4000×3000. The mixed cell images are annotated by several experts as shown in Figure 6. Details of the RBCs, macrophage, and mixed cell images are shown in Figure 7.

## 3. Results

### 3.1. Train Details

In this paper, we ran 35000 training iterations in the Python3.7 environment on an NVIDIA GeForce RTX 2060 GPU with CUDA 10. We used Pytorch for the proposed network training and testing. Similar to [24], the weights in the network are initialized randomly with $\left(\mu ,\sigma \right)=\left(0,\sqrt{2/N}\right)$, where *N* is the number of nodes. In addition, we applied rotation, scaling, and gray value augmentation for improving training results. We then presented the single-kind-cell images processed by image augmentation to the network in mini-batches of size 4 and trained the network with back-propagation using adaptive moment estimation (Adam, *β*_1_ = 0.9, *β*_2_ = 0.999, the learning rate was set as 0.001).

Jaccard index is a metric to compare the similarity and differences between two samples. If the two sets *A* and *B* are given, the definition of Jaccard index is as follows:

(3) $$\begin{array}{c} J\left(A,B\right)=\frac{\left|A\cap B\right|}{\left|A\cup B\right|}=\frac{\left|A\cap B\right|}{\left|A\right|+\left|B\right|-\left|A\cap B\right|}. \end{array}$$

Similar to [25], we use the cross-entropy loss function *H* to punish the classification error of the model and get the final loss function by combining (3) and *H* as follows:

(4) $$\begin{array}{c} L=H-\log\left(J\right). \end{array}$$

By minimizing the loss function, we finally get a 3×512×512 feature map, where each pixel denotes the probability of a class, and each channel signifies the foreground and background (background is 0, RBCs is 1, and the macrophage is 2). We get the final predicted mask images by the pixel value of each location.

### 3.2. Evaluation Metrics

Based on Pont-Tuset and Marques [26], U-Net model can be evaluated with mIoU. The computation of this metric needs 4 values, that is, true positive (TP), true negative (TN), false positive (FP), and false negative (FN). mIoU is calculated as the ratio of TP and (TP + FP + FN), and the formula is

(5) $$\begin{array}{c} mIoU=\frac{1}{k+1}\overset{k}{\underset{i=0}{\sum }}\frac{p_{ii}}{\sum_{j=0}^{k}p_{ij}+\sum_{j=0}^{k}p_{ji}-p_{ii}} \end{array}$$

where *p*_*ii*_ is the number of true positive, *p*_*jj*_ is the number of true negatives, *p*_*ij*_ is the number of false positives, *p*_*ji*_ is the number of false negatives, and *k* + 1 is the number of classes (include background).

Frequency-weighted intersection over union (FWIoU) is an improved IoU that considers each class appearance frequency. It is calculated as

(6) $$\begin{array}{c} FWIoU=\frac{1}{\sum_{i=0}^{k}\sum_{j=0}^{k}p_{ij}}\overset{k}{\underset{i=0}{\sum }}\frac{\sum_{j=0}^{k}p_{ij}p_{ii}}{\sum_{j=0}^{k}p_{ij}+\sum_{j=0}^{k}p_{ji}-p_{ii}} \end{array}$$

where the parameters are the same as (5).

### 3.3. Experiment

Firstly, we verify the importance of each step in the preprocessing algorithm through ablation experiments to demonstrate the effectiveness of our automatic annotation method. A summary of the result can be found in Table 1.

**Figure 8:**
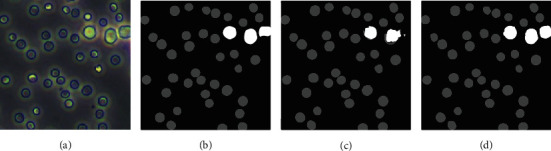
The fine-tuning result of U-Transfer relative to U-Single. (a) Original image. (b) U-Base. (c) U-Single. (d) U-Transfer.

This research examined the performance of the proposed method for U-Net through three experiments. In experiment 1 (U-Base), 600 manually labeled mixed cell images were divided into three parts. Then, we chose 450 images for the training set. Also, 75 images were used for the validation set, and the other 75 were used for the test set. We regard the result of the U-Base as the baseline of the model. In experiment 2 (U-Single), the single-kind-cell images data were processed using the aforementioned preprocessing method. Two random images in two single-kind-cell images datasets (RBCs and macrophages) were fed into the model for training. Based on the training of U-Single, in experiment 3 (U-Transfer), we used 50 annotated mixed-kind-cell images as the training set to fine-tune the model. The tune result of the U-Transfer is shown in Figure 8. In U-Base and U-Single, the batch size was set as 4, the epoch was 200, and the learning rate was 0.001. In U-Transfer, the epoch was 100; the learning rate was 0.0005.

As shown in Figure 8, the model in U-Single wrongly segments some parts of large cells (macrophages) and leads to the cell edge uneven, while these defects will be overcome by the adjustment in U-Transfer. The accuracy of the model in U-Transfer is also improved (e.g., the macrophage in the top-right corner of the mixed-kind-cell image can be correctly identified).

**Figure 9:**
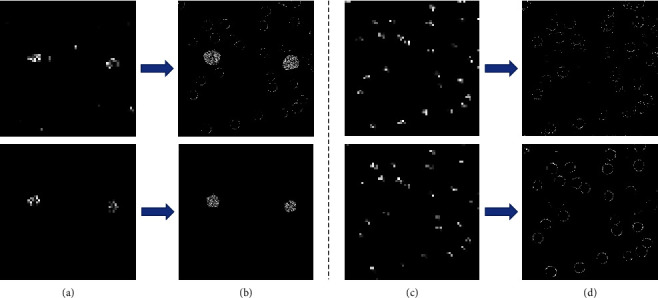
The high-level feature map ((a) macrophages; (c) RBCs) and the low-level feature map ((b) macrophages; (d) RBCs) in the U-Single model (top) and U-Transfer model (bottom).

Figure 9 shows the extracted feature maps of U-Single and U-Transfer, respectively. The feature map of each kind of cell in the fine-tuned U-Transfer model is clearer than that of U-Single. This indicates that the model is more accurate in the recognition of such cells and has better segmentation performance.

**Figure 10:**
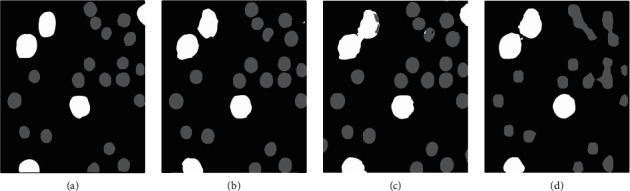
Segmentation masks obtained using different models: (a) ground truth; (b) U-Transfer; (c) TemausNet; and (d) Mask R-CNN.

In order to test the training effect of our proposed method on different models, we used Mask R-CNN [27] and TernausNet [28] as comparative experiments and applied the same training strategy. Mask R-CNN is an improvement of Faster R-CNN [29], since this model added a mask prediction branch that demonstrated competitive performance on instance segmentation. TernausNet replaces the encoder in the U-Net network with VGG11, which contains 7 convolution layers. Each is followed by a ReLU function and 5 max-pooling layers. TernausNet can improve the performance of U-Net by pretrained weights. The comparison results of these models for multiclass segmentation are presented in Figure 10 and Table 2.

The segmentation results of these models illustrate that the proposed method is effective for all of them. Mask R-CNN is good at large object segmentation (macrophages), but it does not accurately do RBCs segmentation. TernausNet has a stronger recognition ability for targets on the image boundary than U-Net, but it cannot distinguish the adjacent objects.

**Figure 11:**
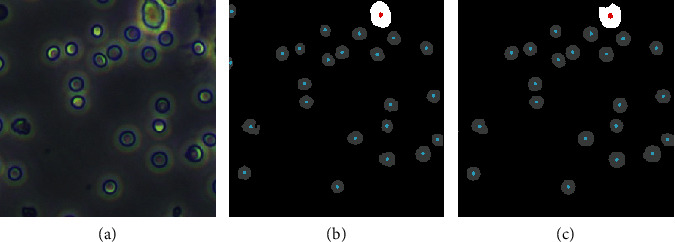
Count results of (a) the annotated mask image and (b) the predicted image.

In addition, we also count the cells according to the training results of the model. The count performance of the model depends on the segmentation result. After excluding the small area, false position (FP), we count the number of cells according to the pixel value and the area. Results are shown in Table 3 and Figure 11. The count results of U-Base and U-Transfer were similar. The counting accuracy of RBCs is low because the RBCs area is small. Also, the RBCs at the image boundary are not easy to be recognized by U-Net, so the U-Transfer model fails to count. While the RBCs segmentation of TernausNet outperforms those from other models, the accuracy of RBCs count is the best in the 4 models.

Considering the biomedical application, we test the running frame rate of the proposed auto-CSC during inference. As for using GPU for acceleration, we realize the auto-CSC in real time at a speed of 512×512 pixels/25FPS. In addition, the FLOPs of the proposed U-Net are 3.09 M, and the params are 7.48 m.

## 4. Discussion

For the image segmentation tasks, the transfer learning should be considered because it is expensive to collect a large volume of training datasets (in particular for medical images) and qualitatively label them. In this paper, we propose a novel framework to segment and count the mixed-kind cell images without too many manual annotations. We train several segmentation models by the proposed method and discuss the changes of the model after fine-tuning. The effectiveness of the method is demonstrated by the training results of 3 different semantic segmentation models. In short, the method we propose here can automatically process cell datasets and train a model to segment cells. This novel methodology can greatly reduce the workload of data annotation without sacrificing the performance of the model. At present, the cell analyzer is commonly used for cell counting, but the accuracy of the cell analyzer is about 90% due to the influence of reagent, temperature, pH, voltage, current, magnetic field, and other factors. And complex preparations are needed before using the cell analyzer. So, the accuracy and speed of our proposed method have reached a satisfactory level.

Besides, our method can be used with more advanced models such as ResNet or LSTM to solve more complex problems [30]. We believe that this new method puts forward a new idea for data processing and lays a solid foundation for the application of deep learning in medical practices in the future.

## 5. Conclusion

In this paper, we propose a novel framework that trains mixed cell images segmentation model by using a small amount of manually annotated cell images. The proposed frame preprocesses the cell image based on the traditional image processing algorithm and uses U-Net for semantic segmentation. It is worth mentioning that the FWIoU of the model is 94.85%, which equals the model trained by large amounts of annotated mixed cell images. In addition, we also realize real-time cell counting by this frame and greatly reduce the workload of doctors. Extensive experiments on mixed cell images datasets demonstrate the superiority and effectiveness of our approach.

## Figures and Tables

**Table 1:** Automatic annotate single-kind cell images results by using preprocessing.

Gaussian filtering?		**√**		**√**
Adaptive thresholding?	**√**	**√**	**√**	**√**
Contour detection?	**√**	**√**	**√**	**√**
Morphological processing?			**√**	**√**
RBCs mIoU (%)	0.7928	0.8142	0.8003	*0.8175*
Macrophages mIoU (%)	0.6356	0.6843	0.6712	*0.6926*

**Table 2:** Performance comparison on different methods.

	U-base	U-single	U-transfer	Mask R-CNN	TernausNet
mIoU (%)	80.47	71.33	**79.73**	65.06	77.58
FWIoU (%)	95.08	92.69	**94.85**	90.89	94.43
Dice score	0.8869	0.8234	**0.8812**	0.7660	0.8678

**Table 3:** The count result of U-Base, U-Transfer, Mask R-CNN and TernausNet.

	Ground truth	U-Base	U-Transfer	Mask R-CNN	TernausNet
RBCs	3130 (100%)	2922 (93.35%)	2925 (93.45%)	2232 (71.31%)	*3045 (97.28%)*
Macrophages	405 (100%)	411 (101.48%)	*401 (99.01%)*	354 (87.41%)	398 (98.27%)

## Data Availability

The data used to support the findings of this study are available from the corresponding author upon request.
